# Systematic review on traditional medicinal plants used for the treatment of malaria in Ethiopia: trends and perspectives

**DOI:** 10.1186/s12936-017-1953-2

**Published:** 2017-08-01

**Authors:** Getachew Alebie, Befikadu Urga, Amha Worku

**Affiliations:** 1 Department of Biology, Jigjiga University, P.O. Box-1020, Jijiga, Ethiopia; 2 College of Veterinary Medicine, Jigjiga University, P.O.Box-1020, Jijiga, Ethiopia

**Keywords:** Medicinal plants, Malaria, Ethiopia

## Abstract

**Background:**

Ethiopia is endowed with abundant medicinal plant resources and traditional medicinal practices. However, available research evidence on indigenous anti-malarial plants is highly fragmented in the country. The present systematic review attempted to explore, synthesize and compile ethno-medicinal research evidence on anti-malarial medicinal plants in Ethiopia.

**Methods:**

A systematic web search analysis and review was conducted on research literature pertaining to medicinal plants used for traditional malaria treatment in Ethiopia. Data were collected from a total of 82 Ethiopian studies meeting specific inclusion criteria including published research articles and unpublished thesis reports. SPSS Version 16 was used to summarize relevant ethno-botanical/medicinal information using descriptive statistics, frequency, percentage, tables, and bar graphs.

**Results:**

A total of 200 different plant species (from 71 families) used for traditional malaria treatment were identified in different parts of Ethiopia. Distribution and usage pattern of anti-malarial plants showed substantial variability across different geographic settings. A higher diversity of anti-malarial plants was reported from western and southwestern parts of the country. Analysis of ethno-medicinal recipes indicated that mainly fresh leaves were used for preparation of remedies. Decoction, concoction and eating/chewing were found to be the most frequently employed herbal remedy preparation methods. Notably, anti-malarial herbal remedies were administered by oral route. Information on potential side effects of anti-malarial herbal preparations was patchy. However, some anti-malarial plants were reported to have potentially serious side effects using different local antidotes and some specific contra-indications.

**Conclusion:**

The study highlighted a rich diversity of indigenous anti-malarial medicinal plants with equally divergent herbal remedy preparation and use pattern in Ethiopia. Baseline information gaps were observed in key geographic settings. Likewise, herbal remedy toxicity risks and countermeasures generally entailed more exhaustive investigation. Experimental research and advanced chemical analysis are also required to validate the therapeutic potential of anti-malarial compounds from promising plant species.

**Electronic supplementary material:**

The online version of this article (doi:10.1186/s12936-017-1953-2) contains supplementary material, which is available to authorized users.

## Background

Malaria remains one of the world’s leading health problems, causing about 429,000 deaths in 2015, the vast majority of deaths (99%) were due to *Plasmodium falciparum* malaria [1]. In that year, most (92%) of the deaths were estimated to have occurred in the sub-Saharan Africa region. Children were particularly affected by the disease with 70% of malaria-caused deaths occurring among the under five-year age group [1, 2]. In Ethiopia, the majority (around 68%) of populations live in areas deemed malarious or potentially malarious [3]. Despite recent improvements in malaria control strategies, the disease remains a major public health problem and a leading cause of outpatient consultations, admissions and death in the country [4, 5].

In recent years, emergence of drug-resistant *Plasmodium* species has exacerbated the health and economic impact of malaria. In particular, *P. falciparum* (the most pathogenic human parasite) has developed resistance to virtually all currently available anti-malarial drugs [6]. Consequently, research for alternative anti-malarial drugs has accelerated over the last two decades [7]. Historically, medicinal plants have been the focus of many researches aimed at discovering alternative anti-malarial drugs in different parts of the world [8]. This has led to the discovery of numerous anti-malarial compounds with significant structural varieties, including quinines, triterpenes, sesquiterpenoids, quassinoids, limnoids, alkaloids, lignans, and coumarins [9].

Around 80% of Ethiopian populations (particularly rural societies) still rely on traditional medicinal plants to fight a number of diseases. This was attributed to high cost of modern drugs, paucity and inaccessibility of modern health services, and cultural acceptability of traditional medicine [10, 11]. Communities inhabiting different localities in the country have developed their own medical plant arsenals and knowledge on their utilization, management and conservation [12]. A large variety of medicinal plants are used as traditional malaria remedy in different parts of Ethiopia [13–17].

Proper documentation of traditional medicine and plants used in the prophylaxis and treatment of malaria constitutes an important task not only in preserving precious indigenous knowledge and biodiversity but also in enhancing community access to and stakes in improvement of malaria control interventions. It is also crucial for stimulating future research on safety and efficacy of medicinal plants and identification of chemical entities that could be developed into new standardized phytomedicines. In contrast, ethno-botanical and ethno-pharmacological research on indigenous anti-malarial plants is still at a rudimentary stage in Ethiopia [18]. Moreover, available research evidence on indigenous anti-malarial plants is highly fragmented, which underscores serious need for systematic compilation and synthesis.

The present systematic review attempted to explore, synthesize and compile ethno-medicinal research findings on anti-malarial plants in Ethiopia.

## Methods

A systematic analysis and review of research literature related to medicinal plants used for traditional malaria treatment in Ethiopia was conducted between April and October 2016.

### Search strategy

A web-based systematic research literature search strategy was employed. Ethno-botanical/ethno-medicinal studies reporting on medicinal plants used for traditional malaria treatment in Ethiopia were gathered by two different search approaches, including:

- Search for unpublished MSc/PhD thesis research reports using Google search engine and local university websites;
- Search for published journal articles using international scientific databases including PubMed, Science direct, Web of Science, Google scholar, AJOL, etc.

Literature search was performed using the following key terms: Ethiopia/Ethiopian plants/Ethiopian medicinal plants/Ethiopian anti-malarial plants, Malaria/Anti-malarial/Anti-malarial plants, Traditional/Traditional knowledge/Traditional Medicine/Traditional medicinal plants, Medicinal Plants/Medicinal herbs, Indigenous/Indigenous knowledge, Plants/Herbal/Medicine/Remedies, Folk Medicine/Folk remedies/Home remedies/Herbal remedies, Ethnobotany/Ethnobotanical, Ethnopharmacology/Ethnopharmacological, Ethnomedicine/Ethnomedicinal, Ethnopharmaceutical, Medico-cultural.

### Screening and criteria

Screening of search outputs was performed in two stages. First, the title and abstract of identified journal articles/theses was overviewed. Thereafter, suitable articles/theses were downloaded and critically inspected for inclusion in the review. Literature screening was based on the following inclusion and exclusion criteria.

#### Inclusion criteria

Published and unpublished ethno-botanical and ethno-medicinal surveys reporting on anti-malarial plant/s, conducted at any time period in Ethiopia

#### Exclusion criteria

The following types of research data were excluded from analysis:

- Data from review articles, historical documents or experimental studies;
- Data from published and unpublished ethno-botanical and ethno-medicinal surveys lacking information on anyone of the following: study areas/localities, informant’s involvement, scientific plant names, and not reporting information about anti-malarial medicinal plants;
- Data from non-open access journal articles or partially accessed (abstract only) articles.

### Data retrieval

Relevant information pertaining to Ethiopian anti-malarial medicinal plants was retrieved using a structured Excel format by directly quoting reported values. In order to provide uniform information on preparation methods of the remedy, the following terms were established, and they signified the respective preparation processes described herein: *Concoction:* mixing/combining different ingredients to make a dish; *Decoction:* boiling the materials and extracting essences or active ingredients; *Infusion:* macerating/soaking the materials in a liquid or water; *Homogenization:* homogenizing ingredients; *Pounding:* grinding, pulverizing, chopping or crushing of ingredients; *Cooking:* preparing food (remedy) for eating by adding ingredients; *Smoking:* burning dry materials and inhaling the smoke; *Bathing/evaporating:* boiling the materials and taking the vapour or steam through intranasal and whole body.

In addition, missed information in some studies, particularly local name and habit of the plants, and misspelled scientific names were retrieved from Natural Database for Africa (NDA), Version 2.0. In case of some research papers lacked geographic locations of the study localities/districts, information was retrieved through direct web (Google) searching.

### Data analysis

All data were entered into Statistical Software Packages for Social Science (SPSS, software version 16.0). A descriptive statistical methods, percentage and frequency were used to analyse ethno-botanical data on reported medicinal plants and associated indigenous knowledge. The results were presented using charts and tables.

## Results

### Overview of ethno-medicinal studies on medicinal plants

Ethno-medicinal studies on plants demand standard procedures for botanical identification and reliable documentation of indigenous knowledge pertaining to plant distribution, management and traditional medicinal use. A total of 82 original ethno-medicinal studies representing ten different regions in Ethiopia were included in this review. Both published and unpublished (M.Sc. and Ph.D. theses) research reports were reviewed. Overall, the reviewed research reports exhibited comparable qualities compared to slightly modified versions of the criteria set by Willcox et al. [19]. Study quality inconsistencies were noted with regard to sampling and number of knowledgeable informants, as well as completeness of herbal remedy recipe, prescription and dosage, side effects, and antidote information reported (Table 1). Current findings reflect potentially important information gaps and need for standardization of ethno-medicinal studies on indigenous medicinal plants in Ethiopia.

**Table 1:** Characteristics of studies on medicinal plants used for the treatment of malaria in Ethiopia

Evaluation parameters	Total number of studies (n = 82)	
	Criterion	Frequency (%)
Paper types	Published article	64 (78.0)
	Unpublished thesis	18 (22.0)
Botanical identification	Plant collected and verified with informant	3 (3.6)
	Voucher specimen in herbarium	18 (22.0)
	Formal identification by botanist	13 (15.9)
	All	44 (53.6)
	None	4 (4.9)
Informants reliability	≥10 informants interviewed	
	Yes	74 (90.2)
	No	8 (9.8)
	≥2 informants mention use of plant for malaria treatment	
	Yes	66 (80.5)
	No	16 (19.5)
	Informant(s) experience of treating malaria	
	Yes	61 (74.4)
	No	21 (25.6)
	Reliable (fulfill all above criteria)	
	Yes	53 (64.6)
	No	29 (35.4)
Researcher reliability	Used same language as informants	
	Yes	78 (95.1)
	No	4 (4.9)
	Recorded Ethno-medicinal information	
	At least PU, PM and AR	15 (18.3)
	Detailed	58 (70.7)
	Poor	9 (11.0)

*PU* part used, *PM* preparation method, *RA* administration routes

### Anti-malarial medicinal plants in Ethiopia

In aggregate, 82 studies identified a total 200 different plant species used in traditional malaria treatments throughout Ethiopia. Additional file 1 summarizes the distribution of the reported plants according to administrative regions and floristic areas of collection. Additional file 2 summarizes the detail of traditional herbal medicine used for the treatment of malaria in Ethiopia.

### Geographic distribution of anti-malarial plants

**Fig. 1:**
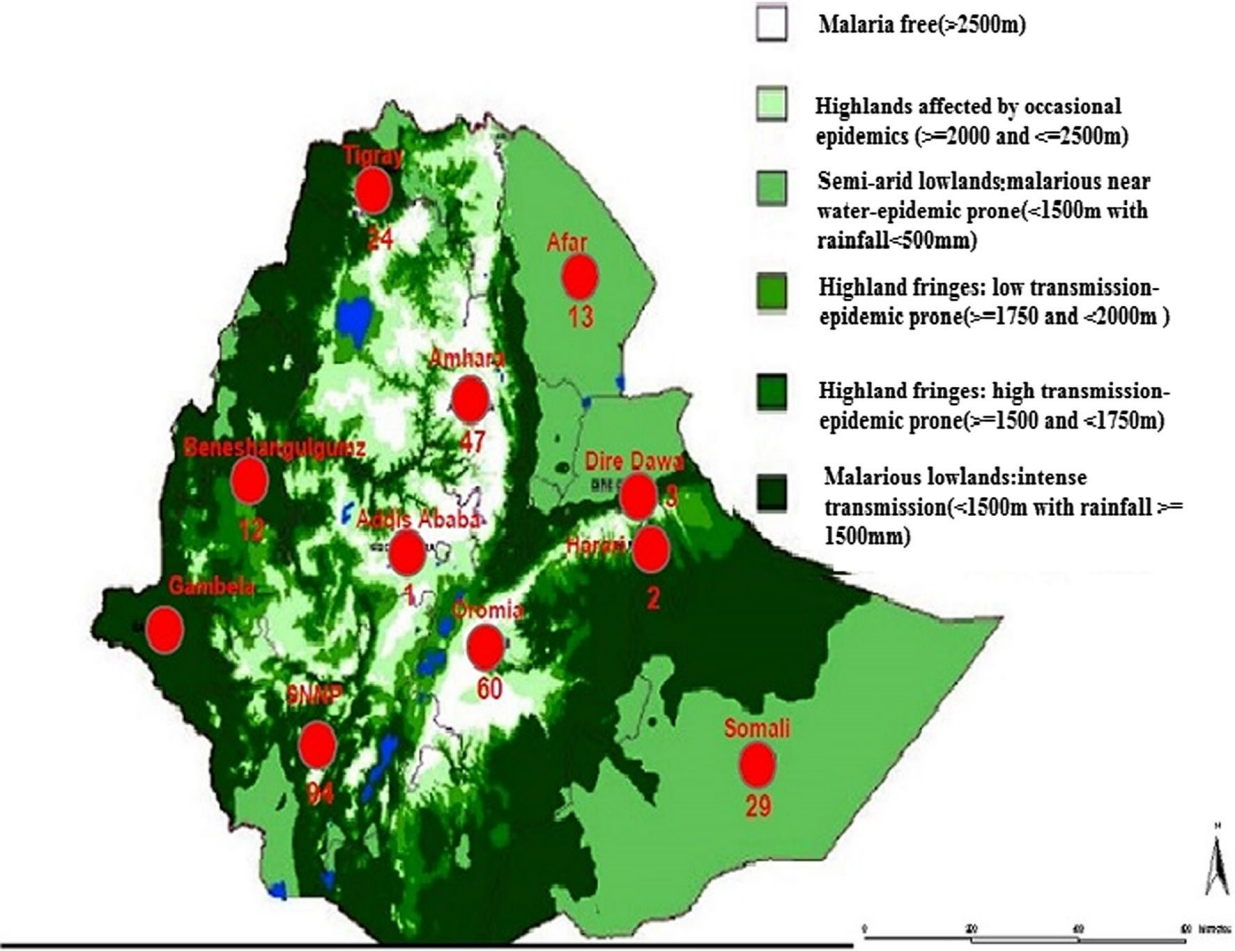
The geographical distribution of anti-malarial plants based on malaria risk stratification map of Ethiopia (adopted from the Malaria NSP 2014–2020). Malaria risk stratification was revised in 2014 using annual parasite incidence per 1000 population (per WHO recommendation) plus altitude and expert opinions from different malaria stakeholders [4]. Malaria risk is thought to be one important factor affecting the abundance of anti-malarial plants. Hence, numbers indicated in the map represent the total amount of anti-malarial plants reported from each administrative region (e.g., 24 plants reported from Tigray region)

The geographic distribution of anti-malarial plants is likely to be predicated on local trend with regard to disease risk, floral diversity and cultural diversity, including traditional medicinal practices. The western lowlands of Oromia, Amhara, Tigray, Southern Nation and Nationality People (SNNP), and almost the entire areas of Benishangul Gumuz and Gambella regions represent the major malarial hotspots in Ethiopia [20]. As shown in Fig. 1, a higher diversity of plants used to treat malaria (94 plant species) was reported from the SNNP region [21–40] followed by Oromia (60) [41–64], Amhara (47) [65–84], Somali (29) [85, 86], and Tigray (24) [87–95] regions. In agreement, others have indicated that medicinal plants were concentrated in southern and southwestern parts of Ethiopia, which possess high biological and cultural diversity [96, 97]. The majority of the plants reported in Amhara (60%) and Oromia (53%) regions were shared by other regions. The Amhara and Oromia regions share boundaries with many other regions in Ethiopia and are likely to share common flora and cultural practices, including in ethno-medicine. Moreover, the limited number of plants reported from highland areas, including Addis Ababa [98] and Harari [99] regions is attributed to zero prevalence of malaria or minimal transmission. Insufficiencies of plants were also reported from the lowland arid regions, including Afar [53, 100, 101] and Dier Dewa [102]. Both regions are characterized by moderate malaria transmission. Despite having rich floral diversity and intense malaria transmission risk, reporting of anti-malarial plants was very low in Benishangul Gumuz [69, 103, 104] and nil in Gambella region (Fig. 1).This may reflect a lack of pertinent ethno-medicinal cultural practices, however, the prevailing gap is probably attributed to serious lapses in ethno-botanical research and documentation of medicinal knowledge and resource in the two regions.

### Diversity of anti-malarial plants

The anti-malarial plant species identified in different region of Ethiopia belonged to 71 different plant families (Additional file 2). Cited plant families included: Fabaceae (18), Lamiaceae (17), Euphorbiaceae (11), Asteraceae (10), Cucurbitaceae and Solanaceae (8 each), Rubiaceae and Aloaceae (6 each), Acanthaceae (5), Moraceae, Brassicaceae and Capparidaceae (4 each), Asclepiadaceae, Anacardiaceae, Apocynaceae, Apiaceae, Malvaceae, Meliaceae, Rutaceae, Ranunculaceae, Rosaceae, Menispermaceae, and Verbnaceae (3 each). The more frequently cited species were: *Allium sativum* (31), *Carica papaya* (20), *Vernonia amygdalina* (18), *Croton macrostachyus* (16), *Lepidium sativum* (15), *Justicia schimperiana* (9), *Phytolacca dodecandra* (8), *Dodonaea angustifolia*, and *Melia azedarach* (7 each), *Clerodendrum myricoides* (6), *Aloe* sp., *Azadirachta indica*, *Brucea antidysenteric*, *Calpurnia aurea* and *Eucalyptus globulus* (5 each), *Ajuga integrifolia*, *Carissa spinarum*, *Artemisia afra*, *Moringa stenopetala*, *Ruta chalepensis*, *Salvadora persica*, and *Tamarindus indica* (4 each). Frequent citation of particular plant species or families could indicate potentially higher bioactive anti-malarial content. Such evidence is pertinent for prioritizing future pharmacological research agendas.

The majority of the anti-malarial plants reported in Ethiopia were shrubs and herbs, 37 and 33.5%, respectively, while tree and climbers was least reported, 23 and 6.5%, respectively. Similar observation was reported in other countries [105, 106]. This trend may be attributed to the abundance and easy access of these growth forms in the country. Others have suggested that shrubs may hold higher content of potential anti-malarial phytochemicals, such as alkaloids and flavonoids [107]. One possible mechanism for the link between shrubs and content of potential anti-malarial phytochemicals could be the diversity and abundance of these plants in different habitats. Secondary metabolites are thought to be required in the adaptation of plants with their environment. In light of this, abundance of shrubs in various habitats could offer a great chance to interact with diverse of biotic and abiotic factors, such as temperature, light intensity, soil nutrients, water supply, herbivore and microbial attack, which might trigger many complex biochemical processes pertaining to synthesize structurally and chemically diverse metabolites with significant anti-malarial activities, including alkaloids and flavonoids.

### Recipe reports

#### Preparation of herbal recipes for malaria treatment

**Fig. 2:**
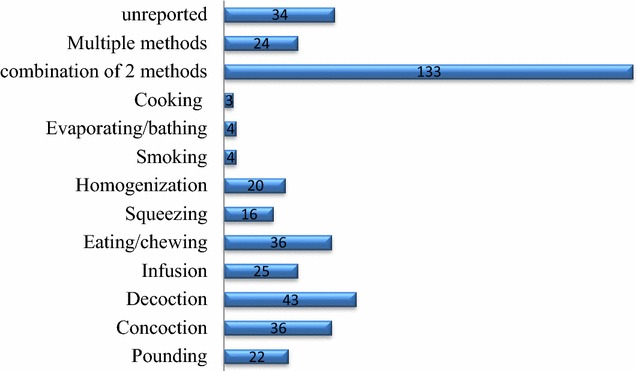
Frequency of herbal preparation methods

Practitioners used either a single method (209) or combinations of two (133) and more (24) methods for preparing anti-malarial herbal remedies. Decoction, concoction, eating/chewing, infusion, and pounding represented the most common independent herbal remedy preparation. Of the herbal remedies prepared by two or more methods, 71.3% were started by pounding or crushing (Fig. 2). Studies from other parts of Africa have also reported that decoction was the most frequently used method of herbal remedy preparation, commonly using water as a solvent [105, 108–111]. Water is a cheaply available solvent that can dissolve a high number of metabolites, and high temperature would permit a rapid extraction of active ingredients. Concoction was also noted as a common method of herbal remedy preparation in Africa [112–114]. This method is believed to enhance synergic effect of medicinal plants and increase the efficacy of herbal remedies. Preference for eating/chewing and pounding/crushing might be related to ease of preparation, and easily available local tools, including stones.

Some of the anti-malarial herbal preparations were prepared from mixtures of two or more different plant species. Notable examples reported in Ethiopia include:

- *Allium sativum* individually combined with one of the following plants; *Girardinia diversifolia* [41]*, Lepidium sativum* [50, 88], *Ruta chalepensis* [87], *Datura stramonium* [50], *Otostegia integrifolia* [72]*, Ocimum basilicum* [45], *Ginger officinale* [45, 50], *Cicer arietinum* [75], *Carica papaya* [29, 50], *Capsicum annuum* [42, 43], *Artemisia afra* [42], *Croton macrostachyus* [56], *Brucea antidysenterica* [65] or with groups of plants such as: *Artemisia afra, Ruta chalepensis* and *Lepidium sativum* [31]; *Solanum dasyphyllum, Lepidium sativum*, *Withania Somnifera, Schinus molle*, and *Sida schimperi* [65];
- *Leucas stachydiformis* with *Ocimum lamiifolium* [49];
- *Maerua oblongifolia* with *Withania Somnifera* [86];
- *Asparagus africanus* with *Aloe* sp. [86];
- *Droguetia iners* with *Premna oligotricha* [39];
- *Rumex abysinicus* with *Zehneria scabra* [69];
- *Silene macrosolen* with *Echinops kebericho* [65];
- *Vernonia amygdalina* with *Ruta chalepensis* [45, 50, 87] or *Carica papaya* [32];
- *Justicia schimperiana* with *Rumex nervosus* and *Vernonia amygdalina* [42];
- *Senna italica* with *Indigofera* sp. or *Zaleya pentandra* [100];
- *Lepidium sativum* with *Echinops kebericho* and *Croton macrostachyus* [31, 49];
- *Salvadora persica* with *Lycium shawii* and *Acalypha* sp. [100];
- *Aloe* sp. with *Asparagus africanus* and *Senna italica* [86];
- *Croton macrostachyus* with *Gardenia lutea* or *Azadirachta indica* and *Carica papaya* [69];
- *Capsicum annuum* with *Otostegia integrifolia, Ocimum gratissimum*, *Prunus persica* and *Schinus molle* [69];
- *Hagenia abyssinica* with *Silene macrosolen, Phytolacca dodecandra*, *Cucumis ficifolius* and *Clerodendrum myricoides* [83];
- *Securidaca longipedunculata* with *Carissa spinarum*, *Capparis tomentosa*, *Withania somnifera* and *Cucurbita* sp. [73].

Aside from anti-malarial plants, various other additives were also used in some herbal preparations. Commonly reported additives include: animal products (egg, meat and milk), honey, sugar, tea, salt, soup, *Eragrostis tef* dough, coffee, lemon, *injera*, local alcoholic drinks (*areke, tella*). Additives were mostly used to moderate the power and/or improve the taste and enhance the efficacy and healing conditions of the remedy [35, 39, 43, 48, 49, 83, 88]. This could possibly be attributed to synergistic effects of the mixtures that might contain a range of pharmacologically active compounds potentially augmenting the chance of the drug interacting with numerous, varied biological targets. Their interaction might influence selectivity, availability, absorption and displacement (distribution) of the remedy, and bioactivity, including enzyme activities. Thus, such traditional practices could provide the opportunity to understand drug interaction and mechanisms of actions, and pave the way to discovering lead structures for the development of novel anti-malarial drugs.

#### Plant parts used and condition of preparations

**Fig. 3:**
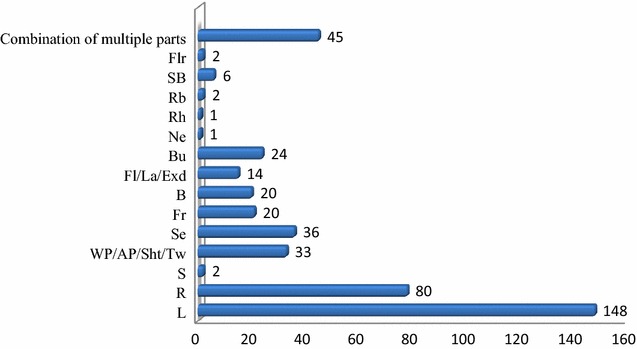
Frequency of the reported plant parts used for herbal preparations

The majority of anti-malarial herbal remedies were prepared from a single plant part while some were prepared from a combination of two or more plant parts. Leaf and root were the most frequently used plant parts (Fig. 3). Leaves were indicated to be the plant parts most commonly used by traditional medicine practitioners in many African countries [115–117]. Leaves are responsible for synthesizing the majority of plant secondary metabolites. This makes them an abundant source of active chemical entities, which can be extracted with relative ease. Regular harvest of leaves poses no/low threat to individual plants survival. This encourages the frequent and safe utilization of leaves in herbal preparations. Plant root structures, such as tuber and rhizome, can be rich sources of potent bio-active chemical compounds. However, frequent usage of roots for herbal preparations can be risky to the survival of a plant species. Therefore, application of proper harvesting strategies and conservation measures is necessary to ensure sustainable utilization of medicinal plant resources.

The majority (62%) of anti-malarial herbal remedies were prepared from fresh plant materials followed by dry (20.9%) and both fresh and dry materials (5%). On the other hand, plant conditions used for this matter are not indicated in 12.1% of the study reports. The predominant use of fresh materials for herbal preparation probably reflects an attempt to capture potent, volatile substances that determine therapeutic efficacy of herbal preparations [118]. Dry materials could be preferred when the plant is poorly accessible. As reported in some of the reviewed studies, some practitioners travel a long distance to collect medicinal plants and practice long-term preservation.

### Routes of administration and dosage of herbal remedies

Anti-malarial herbal remedies were primarily administered through oral route (82.7%), while rarely administered through nasal (5.5%) and whole body (2.8%). Yet, few (9%) reports failed to indicate administration routes of herbal remedies. Liquid herbal preparations made from both fresh and dry materials were taken orally. Fresh solid materials were also eaten and chewed directly upon collection or after initial pounding/crushing. Meanwhile, dry solid materials were smoked and administered through intranasal. These findings were compatible to the observations reported from other countries [112–114]. Malaria is a disease caused by protozoan intracellular haemo-parasites and its treatment entails delivering adequate circulating concentration of appropriate anti-protozoal chemicals. The oral route is a convenient and non-invasive method of systemic treatment. The route permits relatively rapid absorption and distribution of active chemical compounds from herbal remedies, enabling the delivery of adequate curative power [88].Potential risk of enzymatic breakdown and microbial fermentation of active chemical entities may necessitate alternative routes of herbal remedy administration.

Herbal remedy dosage was basically determined by edibility of the plant parts used. In case of remedies prepared from non-edible plants/parts, dose was prescribed based on age, physical strength and health status of patients. However, full dosage determination varied from healer to healer. Variations were noted in the measurement units used for dose estimation, and in the frequency and duration of herbal treatment prescribed. Dose of herbal preparations was usually estimated using different locally available materials/means, including plastic/glass/steel cups (could be coffee-cup, teacup, water-cup) or gourd utensils, number of drops for liquid materials; teaspoons for powders; counting the number of units for seeds, leaves and fruits; index finger estimation of root size. Generally, recommendation was made to administer the herbal remedies twice or three times per day for one, two or three consecutive days to many months or until recovery. Lack of precision and standardization is widely acknowledged to be an important drawback of traditional healthcare systems [119–122].

### Adverse effects, antidotes and contra-indications

In settings where traditional medicine is keen, the pharmacological effect of medicinal plants is generally ascribed to their active and ‘safe’ content that will only exert quick effect when taken in large quantities. However, the majority of the reviewed reports made no mention of possible side effects to different herbal preparations. Nevertheless, herbal preparations made from some anti-malarial plants were reported to have side effects, such as vomiting, nausea, diarrhoea, headache, urination, heartburn, and nightmare [22, 31, 43, 54, 67, 68, 71, 75, 86, 87, 100]. This may be attributed to different underlying factors, including improper dosing, toxic plant chemicals, toxic metabolic byproducts, etc. *Teff injera* and porridge, *Shiro wot* (pulse grain sauce), coffee, milk and milk products, honey, *Shoforo* (infusion made from coffee peel), *Tela,* barley soup and juice of *Sansevieria ehrenbergii* were reported as antidotes for potential herbal remedy side effects [22, 31, 43, 54, 67, 68, 71, 75, 87]. Some anti-malarial plants were reported as contra-indicated to the elderly, pregnant women, children, physically weak persons, and patients with hepatitis [31, 42, 43, 54, 66–68, 71, 75, 86–88, 100] (Table 2). Current observations indicate existence of critical research-evidence gaps with regard to the potential toxicities and corresponding counteracting mechanisms of anti-malarial plants in Ethiopia. This gap represents an important roadblock to effective development and exploitation of indigenous medicinal plant resources.

**Table 2:** Side effects, antidotes and contra-indications of some plants used for traditional malaria treatment in Ethiopia

Species	Side effects	Antidotes	Contra-indication
*Croton macrostachyus*	Diarrhoea, vomiting, headache, urination	*Teff injera* and porridge	Pregnant women
*Hagenia abyssinica*	Vomiting and diarrhoea	*Shiro Wot*	
*Aloe* sp.	Nausea, vomiting, diarrhoea	–	Pregnant women
*Euphorbia abyssinica*	Headache, diarrhoea, vomiting	Coffee and milk, red *teff* porridge, a lot of *aguat*	Pregnant women
*Anethum graveolens*	Vomiting, nausea, diarrhoea	–	–
*Asparagus africanus*	Vomiting, nausea, diarrhoea	–	–
*Aloe otallensis*	Vomiting and diarrhoea	Honey	–
*Cadaba farinose*	Headache	*Shoforo*	–
*Canthium pseudosetiflorum*	Vomiting	*Shoforo*	–
*Cyperus distans*	Nausea and vomiting	*Shoforo*	–
*Hypoestes forskaoli*	Headache, heartburn, nausea/vomiting and nightmare	Juice of *Sansevieria ehrenbergii*	–
*Phytolacca dodecandra*	Vomiting and diarrhoea	Milk, red *teff* porridge, coffee, *Tela*	Children, pregnant women, patient with hepatitis
*Justice schimperiana*	Vomiting and diarrhoea	Milk, red *teff* porridge	Children, pregnant women
*Andrachne aspera*	–	–	Pregnant women
*Gnidia involucrata*	–	–	Patient with hepatitis, babies/old people, pregnant women
*Ajuga integrifolia*	Vomiting and diarrhoea	Boiled coffee, milk or barley soup	Pregnant women, physically weak person

### Trends in anti-malarial plant research and development

In different African countries, many of the anti-malarial plants identified in this paper have demonstrated promising therapeutic potential on pre-clinical and clinical investigations. Notable examples were *Artemisia annua* [123, 124], *Ajuga remota* [125], *Azadirachta indica* [126–128], *Argemone mexicana* [129, 130], *Vernonia amygdalina* [131–135], *Asparagus africanus* [136], *Uvaria leptocladon* [137], and *Gossypium* spp. [138]. In parallel, multiple promising candidate anti-malarial compounds have been identified from these plant resources [139–145]. Consequently, international market demand (Switzerland, France, China, etc.) for African medicinal plants has exhibited sustained growth. Export of promising indigenous medicinal plant resources offers substantial contribution to the economy and growth of African countries. For instance, export of traditional medicines contributed an estimated R2.9 billion to South Africa’s economy [146]. Likewise, Egypt’s 2008 exports of selected medicinal plants amounted to 77,850,312 kg with a reported value of US $174,227,384 [147].

Despite the remarkable historic success of traditional medicinal practices and abundance of indigenous medicinal plant resources (Additional file 1), anti-malarial ethno-pharmacological research in Ethiopia remains at primitive stage, with scope limited to evaluating crude extracts from various anti-malarial plants against *Plasmodium berghei*. A prominent gap is evident with regard to research geared towards identifying plant bioactive entities, and establishing the efficacy and safety of medical plants through in vitro assays using human *Plasmodium* parasites, in vivo assay involving higher animal models and randomized clinical trials. Absence of favourable medicinal plant research and development impedes optimum exploitation of potential economic benefits. Thus, despite holding one of the richest (diversity and quantity) resources in the continent, large-scale production and export of medicinal plants has remained limited in Ethiopia. Prevailing scenarios underscore a pressing need for enhancing pre-clinical and clinical research aimed at developing safe, effective and affordable alternative anti-malarial agents from indigenous plant resources. This requires collaborative engagement involving government bodies, researchers, traditional healers, and prospective business investors.

## Conclusion

The study highlighted that a rich diversity of indigenous medicinal plants were commonly used for traditional treatment of malaria in Ethiopia. Ethno-medicinal research on distribution and usage pattern of anti-malarial plants shows substantial variability across a spectrum of geographic and social strata in the country. Baseline information gaps are evident in key geographic settings, such as the Beshangul Gumuz and Gambella regions. Divergent preparation and use patterns of anti-malarial herbal remedies, as well as associated toxicity risks and countermeasures, generally demand deeper, exhaustive investigations. Experimental research and advanced chemical analysis are required to identify and validate the therapeutic potential of anti-malarial chemical compounds from promising plant species, with due consideration to efficacy and safety issues. Sustainable development and exploitation of indigenous medicinal plant resources entails coordinated multidisciplinary research programmes that give due credit to traditional practitioners and engage with commercial investors.

## Additional files

**Additional file 1.** Distribution of the reported medicinal plants used for the treatment of malarial based on administrative regions, and floristic areas of collection confined.

**Additional file 2.** Medicinal plant families, species, local name, habit, parts used, preparation methods and other medicinal values of plants used for the treatment of malaria in Ethiopia.
