# Supplementary material for: Systematic review on traditional medicinal plants used for the treatment of malaria in Ethiopia: trends and perspectives
Source: Malar J. 2017 Aug 1;16:307. doi: 10.1186/s12936-017-1953-2 (PMC5540187; doi:10.1186/s12936-017-1953-2)
Supplement: Supplementary file 1 — Additional file 1. Distribution of the reported medicinal plants used for the treatment of malarial based on administrative regions, and floristic areas of collection confined. [file 12936_2017_1953_MOESM1_ESM.doc]

**Additional file 1:** Distribution of the reported medicinal plants used for the treatment of malarial based on administrative regions, and floristic areas of collection confined

| ***Administrative region (total no. of plant spp.)*** | ***Frequency of reported plant Species (%)*** | | | ***Floristic areas of collection*** |
| --- | --- | --- | --- | --- |
|  | ***No. of Plant/s confined to respective region*** | ***Plant/s shared with other regions*** | | ***Location:*** ***Districts/weredas*** |
|  |  | ***Regions (no. of shared plant/s)*** | ***Total shared plants*** |  |
| Addis Ababa **(n=1)** | 0(0.0) | G & I(1) | 1(0.4) | ***CE:*** *Akaki* |
| Afar **(n=13)** | 6(2.1) | D , H & I (1); G & H (1); H (2); G (1); H & I (1); D, G & H (1) | 7(2.4) | ***NEE:*** *Chifra;Aba'ala;Awash National Park* |
| Amhara **(n=47)** | 19(6.6) | H (1); G, I & J (4); E, G & H (1); G & I (4); I (6); G (5); J (2); D, G, I & J (1); E & J (1); D, G & I (1); I & J (1); G, H & I (1) | 28(9.8) | ***CE:****Debre libanos, Debre Libanos monastery,*  ***NCE:****Ankober, North Shoa Zone*  ***NWE:*** *Bahirdar zuria, Chagni, Guangua, Farta, Jabitehnan, Mecha, Gozamen,Zegie Peninsula, Libo Kemkem, Chilga, Gondar*  ***NE:****Berehet,Dega Damot, Debark,Wogera (Ambagiorgis area), Delanta,*  ***SEE****:Minjar-Shenkora* |
| Benshangul- Gumuz **(n=14)** | 7(2.4) | B, H & I (1); I & J (1); C, G, I & J (1); I (1); G, I & J (1); C, G & I (1); B, G & H (1) | 7(2.4) | ***WE:****Mandura*  ***Mid-WE:****Menge and Komehsa (Berta ethnic group)*  ***NWE:****Dibatie* |
| Dire Dawa **(n=3)** | 0(0.0) | C, G & H (1); C & J (1); I (1) | 3(1.0) | ***EE:****Harla and Dengego valleys* |
| Harari **(n=2)** | 0(0.0) | G (1); G, H & I (1) | 2(0.7) | ***EE:****Harar town* |
| Oromia **(n=60)** | 28(9.8) | H (1); A & I (1); C, I & J (4); I (7);B & H (1); C, E & H (1); B (1); C & I (4); C (5); C, D, I & J (1); G, I & F (1); D, I & J (1); F (1); C, D & I (1); B, D & H (1); C, H & I (1); | 32(11.1) | ***CE:****Fiche town market,Around Fiche District*  ***CEE:****Boosat,Semi-Arid East Shewa(Boosat & Fentallie), Fentallie,*  ***WE:****Gindeberet, Sasiga,Horro Gudurru, Ejaji Area, Wayu Tuka,Jima Rare*  ***SWE:****Jimma zone(Kersa, Manna, Gomma, Dedo, Limmu Genet, Limmu Seka)Ghimbi,Goma, Seka Chekorsa, Asendabo*  ***SEE:****Delomenna, Dheeraa, Mana Angetu*  ***EE:****Erer Valley of Babile;*  ***NEE:****Awash National Park*  ***SE:*** *Abaya(Borana), Blue Hora* |
| Somali  **(n=29)** | 16(5.6) | C (1); G (1); J (1); B, D & I (1); B & G (1); C, E & G (1); B (2); G, I & F (1); I (1); B & I (1); B, D & G (1); C, G & I (1) | 13(4.5) | ***EE:*** *Shinile; Jgjiga* |
| SNNP  (Southern Nation and Nationality  Peoples )  **(n=94)** | 59(20.6) | A & G (1); C & G (7); B, D & G (1); G (7); D & J (1); C (5); C, D & G (2); D (1); G, H & F (1); D, G & J (1); J (1); E (1); C, G & J (2); H(1); C & J (1);B & H (1); C, G & H (1) | 35(12.2) | ***SE:****Boricha, South Omo, lower Omo valley (Kara and Kwego), Hamer,Amaro Special District, Benna Tsemay, Wondo Genet, Hawassa, Dawuro, suri,Chencha,Wonago, Mirab-Badwacho, Kembatta Tembaro, Hadiya Zone*  ***SWE:****Bench, Sheko,Meinit Goldya and Meinit-Shasha, Konso*  ***SEE:*** *Kochere* |
| Tigray **(n=24)** | 11(3.8) | C, G & I (4); C (2); C, D, G & I (1); C & E (1); C & I (1); D, G & I (1); H (1); D & I (1); I (1); | 13(4.5) | ***NE:****Gemad, Kilte Awulaelo, Endrta District, Ofla,Seharti Samre,Tahitay Adiyabo and Kafta Humera(Kunama ethnic), Asgede Tsimbila, Central Zone of Tigray, degraded dry lands of Tigray* |
| **Total (287)** | **146(50.9)** |  | **141(49.1)** |  |

A=Addis Ababa, B=Afar, C=Amhara, D=Benishangul Gumuz, E=Dire Dawa, F=Harari, G=Oromia, H=Somali, I=SNNP, J=Tigray; **_NEE_**_:North East Ethiopia_, **_CE_:** _Central Ethiopia_, **_CEE_:** _Central East Ethiopia_, **_NWE_:** _North West Ethiopia_, **_NE_:** _Northern Ethiopia_, **_NCE_:** _North Central Ethiopia_, **_WE_:** _West Ethiopia_, **_Mid-WE_:** _Mid-West Ethiopia_, **_EE_:** _Eastern Ethiopia_, **_SE_:** _Southern Ethiopia_, **_SWE_:** _South West Ethiopia_, **_SEE_:** _South East Ethiopia_

The above table showed the total number of anti-malarial plants reported from each administrative region. But plants in the respective region can be reported either only from that region or also other regions [for example a total of 1 plant species was reported from Addis Ababa, but the plant also reported from Oromia and SNNP region; 13 plant species were reported from afar region, but 6 of which plants are confined to Afar while the remaining 7 plants are shared with group of regions including with Somali, Benishangul Gumuz and SNNP region(1 plant); Oromia and Somali (1 plant); Somali (2); Oromia (1); Somali and SNNP (1); Somali, Benishangul Gumuz and Oromia (1)]. In addition, the table indicates floristic area of collection within each region (for example, plant reported from Addis Ababa was collected in Akaki area of the region; plants reported from Afar were collected in different districts/areas of this region, namely Chifra, Aba'ala, and Awash).
