# Supplementary material for: Systematic review on traditional medicinal plants used for the treatment of malaria in Ethiopia: trends and perspectives
Source: Malar J. 2017 Aug 1;16:307. doi: 10.1186/s12936-017-1953-2 (PMC5540187; doi:10.1186/s12936-017-1953-2)
Supplement: Supplementary file 2 — Additional file 2. Medicinal plant families, species, local name, habit, parts used, preparation methods and other medicinal values of plants used for the treatment of malaria in Ethiopia. [file 12936_2017_1953_MOESM2_ESM.docx]

Additional File 2: Medicinal plant families, species, local name, habit, parts used, preparation methods and other medicinal values of plants used for the treatment of malaria in Ethiopia

| **Family** | **Scientific name** | **LN** | **Ha** | **PU** | **Methods of preparation(References)** | **Other medicinal values** |
| --- | --- | --- | --- | --- | --- | --- |
| Acanthaceae | *Hypoestes forskaolii* (Vahl) R.Br | Chikecho(Sd) | H | L | Homogenization[21] | Evil eye, dysentery, stomachache, TB/coughing with blood, headache, nausea/vomiting, nightmare |
|  |  |  |  | R | Pounding, infusion and concoction[22] |  |
|  |  |  |  | B | Decoction [23] |  |
|  | *Justicia ladanoides* Lam. | Kakma(Am) | Sh | L | Cooking) [103] | Headache and stomachache |
|  | *Justicia schimperiana* (Hochst.ex Nees) T. Anders | Sensel (Am) | Sh | L | Concoction [24, 25, 41, 42], squeezing, infusion[26, 66], pounding, squeezing and concoction [65] | Intestinal parasites, dysentery, Jaundice, wound, arthritis, gonorrhea, rabies, headache, tooth ache |
|  |  |  |  | Sht | Infusion [67] |  |
|  |  |  |  | L& Sht | Pounding, decoction [68] |  |
|  | *Crabbea velutina* S.Moore | Malgissa (Ko) | H | L | Pounding and infusion[23] |  |
|  | *Acanthus polystachyus* Delile | Dendero (Am) | H | R | Chewing [69] | Intestinal worms, trachoma, wound, retained placenta |
| Aizoaceae | *Zaleya pentandra* (L) Jeffrey | Urribaqla (Af) | H | WP | Pounding and infusion [100] | Snakebite |
| Alliaceae | *Allium cepa* L. | Key-shinkurt (Am) | H | Bu | Chewing[70] | Asma, eye diseases, taeniasis |
|  | *Allium sativum* L. | Nech shinkurt  (Am),  Tuma(Sd) | H | Bu | Infusion, homogenization [21, 65], pounding [27, 48,49, 50], chewing [28], pounding/homogenization [29, 30, 65, 66, 76, 89,90**,** 103] concoction [41, 42, 43, 44, 71, 72, 87,72] , pounding/concoction[45, 88], eating [46, 47, 73,74], pounding/infusion[75] | Tonsillitis, abdominal pain, wound, cough, paralysis, toothache, amoebasis, rabies, *“Mich”*, headache, skin disease, snake bite, lung abscess, paralysis, leishmaniasis, asthma, heart failure, hemorrhoides, gonorrhea, typhoid, neumonia, evil eye, gastritis, ring worm |
|  |  |  |  | S/L | Chewing or pounding [21, 32] |  |
|  |  |  |  | Fr | Chewing [31] |  |
| **Family** | **Scientific name** | **LN** | **Ha** | **PU** | **Methods of preparation(References)** | **Other medicinal values** |
| Aloaceae | *Aloe camperi* Schweinf. | Ureita(Af) | Sh | L | Squeezing [101] | Bleeding, conjunctivitis |
|  | *Aloe macrocarpa* Tod. | Algae (Sd) | Sh | La | Squeezing [29] | Gastritis, emaciation |
|  | *Aloe megalacantha* Baker | Argeesa (Am), Dacar(So) | Sh | L | Pounding, concoction[85],  Squeezing and infusion [88] | Wound, intestinal parasites, snake bite, urine retention, evil eye, impotence, diabetes, dandruff, |
|  |  |  |  | Exd | Concoction[87] |  |
|  | *Aloe otallensis* Baker | Welqante(Ham) | Sh | Exd | Concoction[22] | Wound healing |
|  | *Aloe pirottae* Berger | Hargeysa(Or), Gebedherta(So) | Sh | La | Squeezing, Concoction [51] | Tropical ulcer colon, eye disease, snake bite, gallstone |
|  | *Aloe sp.* | Argessa(Sd) | Sh | L | Homogenization [21], Squeezing, concoction and Homogenization [86], eating [100],  Squeezing and homogenization [100] | Burn, wounds , asthma, bloat , vomiting, diarrhea, stomachache, muscle cramps, TB, diabetes |
|  |  |  |  | Exd | Squeezing and concoction [22, 69] |  |
| Amaranthaceae | *Pupalia micrantha* Hauman | Deg deg (So) | Sh | R /RB | Decoction[86] |  |
|  | *Achyranthes aspera* L. | Telenge (Am) | H | WP | Pounding, squeezing and homogenization [52, 77] | Gonorrhea, snake bite, blood clotting, tonsillitis, abdominal pain |
| Anacardiaceae | *Ozoroa insignis* Del. | Shelel (Am) | T | B | Pounding and infusion [22] |  |
|  | *Rhus natalensis* Bernh. ex C.Krauss | Mst-Aybelash (Am) | Sh | L | Decoction[28] | Cough |
|  | *Schinus molle* L. | Tikur-berbere (Am) | T | Se | Eating [47], pounding and homogenization[65], pounding [69] | Jaundice, diarrhea, tonsillitis, *“Mich”*, heartache, tuberculosis, abdominal cramp/colic |
| Anonaceae | *Uvaria leptocladon* Oliv. | Zebko(Ka) Chochum (Kw) | T | R | Pounding/decoction [33] | TB, coughing with blood, swelling, dysentery, stomachache, abscess, weakness |
| Apiaceae | *Anethum graveolens* L. | Ensilal (Am) | Sh | L&R | Pounding and decoction [86] | Amenorrhea, angina, asthma, body odor |
|  | *Foeniculum vulgare* Miller | Ensilal (Am) | H | R | Decoction [86] | Gonorrhea, stomach ache, coughs, dysentery, abdominal pain, Anuria |
|  | *Carum copticum* L. | Tikur-azmud (Am) | H | Se | Concoction [44] |  |
| **Family** | **Scientific name** | **LN** | **Ha** | **PU** | **Methods of preparation(References)** | **Other medicinal values** |
| Apocynaceae | *Acokanthera schimperi* (A.D.C.) Schweeinf. | Merenz (Am) | T | L | Concoction[51, 102],  pounding and infusion[86] | Skin disorders, hemorrhoids, hepatitis, wound, gonorrhea, Jaundice, mental disorders, tonsilitis |
|  | *Lactuca glandulifera* Hook.f. | Maracha (Sd ) | H | AP | Infusion [21] |  |
|  | *Carissa spinarum* L*.* | Kirketcho (Sd), Agam(Am) | H | R | Homogenization [21], pounding and infusion [22], decoction [34], pounding, concoction and smoking [73] | Evil eye, snake bite, impotence gonorrhea, stomachache, headache, jaundice, muscle cramps, bleeding, swelling of/sore throat, mental disorder, injury |
|  |  |  |  | Fr | [78] |  |
| Arecaceae | *Phoenix reclinata*Jacq. | Meexxi(Or) | T | S, R | Pounding and squeezing[47] |  |
| Asclepiadaceae | *Leptadenia hastata* (Pers.) Decne. | Mesker (So) | Cl | R /RB | Decoction[86] | Wounds, headache, abdominal complaints, urethral discharge, gonorrhoea, diarrhoea |
|  | *Balanites aegyptiaca* (L.) De. | Bedeno (Am) | Sh | L | Concoction [51], homogenization[53], | Anthelmentic, leucoderma, herpes, wounds, syphilis, rheumatism, *“Mich”*, hypertension, typhus, madness, snake bite, influenza |
|  | *Calotropis procera* (Ait.) Ait. f. | Yahara-zaf (Am) | Sh | La,  RB | Squeezing or pounding, homogenization  [54] |  |
| Asparagaceae | *Asparagus africanus* Lam. | Yeset Qest(Am) | Sh | Fr | [78] | Dislocated bone, leshimaniasis, wound, abdominal pain, toothache, skin lesion, evil eye, rabies |
|  |  |  |  | L, R | Pounding and infusion[86] |  |
| Asteraceae | *Vernonia adoensis* Sch. Bip. ex Walp. | Feres-zeng, (Am) | Sh | L | Decoction and concoction[104] | Menstrual disorders, abdominal complaints, *“Mich”*, evil eye, hypertension, snake bite, worm, gastritis, wound |

| **Family** | **Scientific name** | **LN** | **Ha** | **PU** | **Methods of preparation(References)** | **Other medicinal values** |
| --- | --- | --- | --- | --- | --- | --- |
| Asteraceae | *Vernonia amygdalina* Del. | Grawa(Am),  Birmayda (Kr) | Sh | L | Homogenization[21, 42], decoction/ concoction[21, 24, 87], pounding /infusion [27], pounding/ homogenization [28,29, 31, 45,50], infusion [43, 67], squeezing[49,55] | Toothache, abdominal pain, snake poison, ascariasis, hepatitis , tonsillitis, headache, wound, goiter, *“mich”*, diarrhea, heart problem, dandruff, gastritis, cough, urinating problem, eczema, retained placenta, |
|  |  |  |  | L, R&B | 23, [54] |  |
|  |  |  |  | Sht | Pounding / concoction [32] |  |
|  |  |  |  | R | Infusion[35, 67] |  |
|  | *Vernonia auriculifera* Hiern | Regicho(Sd) | Sh | L | Decoction[21, 44] | Toothache, snake poison, eye diseases, |
|  | *Vernonia bipontini* Vatke | Gurbi (Or | Sh | B&L | [59] | Purgative, venereal disease, snake bite, vermifuge |
|  | *Vernonia sp.* | Heten(Gu) | Sh | L | Decoction and concoction [104] | Diarrhea |
|  | *Artemisia abyssinica* Sch. Bip. ex A. Rich. | Chiqugn (Am) | H | L | Pounding and infusion [27] | Ghoneria, leprosy, cough, syphilis |
|  | *Artemisia afra* Jacq. ex Willd. | Chiqugn (Am) | H | L | Pounding and concoction [24, 31, 42, 56] | Abdominal pain, headache, constipation |
|  | *Artemisia annua* L. | Chiqugn (Am) | S | Fr/L | Pounding and squeezing [35] |  |
|  | *Conyza pyrrhoappa* Sch.Bip. ex A. Rich. | Dadaho(So) | Sh | L, S | Bathing [86] | - |
|  | *Echinops hoehnelii* Schweinf. | Qeber(Shk) | H | R | Chewing [36] fumigating [103] | Snake bite, eye infection |
|  | *Echinops kebericho* Mesfin | Kebericho(Am) | H | R | Pounding [79] | Common cold, epidemics |
| Balanitaceae | *Balanites rotundifolia* (van Tieghem) Blatter | Kuzo, Kulan (Am) | Sh | L | Decoction [86], pounding and infusion [100], pounding and squeezing [101] | Dermatophilosis, mange mites, diarrhea, asthma, cough, wound |
| Bignoniaceae | *Stereospermum kunthiamum* Cham. | Botoro(Or) | T | S | Pounding and squeezing [47] |  |

| **Family** | **Scientific name** | **LN** | **Ha** | **PU** | **Methods of preparation(References)** | **Other medicinal values** |
| --- | --- | --- | --- | --- | --- | --- |
| Boraginaceae | *Cordia Africana* Lam. | Wanza(Am) | T | R & B | Decoction [28] | Jaundice, dental problem, diarrhea, jaundice |
|  |  |  |  | SB | Decoction [71] |  |
|  |  |  |  | Fr | [78] |  |
|  | *Cynoglossum coeruleum* Hochst | Batartusa(Sd), Shimgigit(Am) | H | AP | Infusion [21] | Burning, *“Mich*’, amoeba, toothache, bone setting |
| Brassicaceae | *Lepidium sativum* L. | Feto, Heto (Sd) | H | Se | Homogenization [21], decoction [23, 42, 66],  pounding and concoction [28,31, 47,50, 56, 65], pounding [43,87], smoking [49] | Pancreas disease, intestinal parasites, *“Mich”*, headache, diarrhea, wound, swelling, haemorrhoids, evil eye, heartache, leg distortion, allergic reaction, gland TB, cough, tonsilitis |
|  |  |  |  | Fr | Infusion [54, 80] |  |
|  | *Brassica carinata* A.Br | Gomen (Am) | Sh | Se | Pounding [29] | Common cold |
|  | *Brassica nigra* (L.) Koch. | Senafch(Am) | Sh | Se | Roasting, pounding, infusion, fermenting and concoction [81] |  |
|  | *Brassica oleracea* L. | Shena(Sd ) | Sh | Se | Homogenization [21] | Stomach burn |
| Burseraceae | *Boswellia papyrifera* (Del.) Hochst | Etan(Am) | T | Gm | [91] |  |
|  | *Commiphora Africana* (A.Rich.) Endl. | Anqua(Am) | Sh | R & RE | Decoction [28] | Skin irritation |
| Campanulaceae | *Lobelia sp.* | Jibira(Am) | Sh | R | Chewing [69] |  |
| Canellaceae | *Warburgia ugandensis* Sprague. | Kanafa (Am), Bifti (Or) | T | B | Pounding, concoction [57] | TB, bronchitis, pneumonia, hepatitis, tapeworm, stomach ache, gonorrhea, and asthma |
| Capparidaceae | *Maerua oblongifolia* (Forssk.) A. Rich. | Wawatie (Am) | Sh | L | Pounding and decoction[86] | Wound dressing, dental sticks |
|  | *Boscia coriacea* Pax | Shodo (Ka, Kw) | T | R | Pounding/decoction [33] | Diarrhea, thinning, ear pain |
|  | *Cadaba farinosa*  Forssk | Dngay-Seber (Am) | Sh | R | Pounding and decoction [22] | Hypertension, diarrhea, stomachache, headache |
|  |  |  |  | L | Pounding and squeezing [34] |  |
|  | *Capparis tomentosa* Lam*.* | Gombor (Or)  Gimero (Am) | Sh | L | Pounding, infusion [52, 77] | Epidemic |
|  |  |  |  | R | Pounding, concoction, and smoking[73] |  |
| **Family** | **Scientific name** | **LN** | **Ha** | **PU** | **Methods of preparation(References)** | **Other medicinal values** |
| Caricaceae | *Carica papaya* L. | Papaya (Am) | T | L | Pounding[24, 30, 46**,** 57], chewing [26,37,69], decoction[29,32,43,44,50, 58, 74], squeezing[47, 49, 82], concoction[75] | *“Mich”*”, intestinal parasite, diarrhoea, wound, gastritis, anemia, evil eye, cough, stress, epidemics |
|  |  |  |  | Se | Pounding[27, 66] |  |
|  |  |  |  | R | Decoction[49], Chewing, [69] |  |
| Caryophyllaceae | *Silene macrosolen* Steud*.* ex A. Rich | Woggert (Am) | H | R | Pounding and smoking[65], Smoking [88] | Evil spirits, taeniasis, ascariasis, cutaneous leishmaniasis, fibril illness |
| Celastraceae | *Maytenus arbutifolia* (A.Rich.) Wilc | Borbodich(Sd) | Sh | R | Chewing [21] | Itching/scabies, psychiatric disease, wound, |
| Chenopodiaceae | *Halothamnus somalensis*  (N.E.Br.) Botsch. | Mirgi-edalis (So) | Sh | R/ RB | Pounding and decoction [86] | Internal parasites |
| Combretaceae | *Combretum molle* R. Br. ex G. Don. | Abalo (Am) | Sh | L & B | Decoction [28] | Back pain, evil eye, stomach pain, wounds |
|  |  |  |  | L | Decoction [83] |  |
|  | *Terminalia brownie* | Merassa (Ku), Sebaea (Tig) | T | B | Pounding, homogenizing, infusion [92] |  |
|  | *Anogeissus leiocarpa* (DC.) Guill. & Perr. | Mokk(Am) | T | B/L | [93] | Stomach pain, diarrhoea, wounds |
| Convolvulaceae | *Ipomoea kituiensis* var. massaiensis Verdc. | Laalata(Kr) | Cl | L | Concoction [28] | Swelling |
| Crassulaceae | *Aeonium leucoblepharum* Webb ex A. Rich*.* | Yefeyel-Dabo (Am) | H | Bu | Concoction[76] | Atopic eczema, ringworm, asthma, dandruff, pneumonia, coughing, hepatitis |
|  | *Kalanchoe petitiana* A. Rich. | Amdohale(Or) | H | WP | [59] | Ghonnerhia, syphilis, trachoma, tapeworm |

| **Family** | **Scientific name** | **LN** | **Ha** | **PU** | **Methods of preparation(References)** | **Other medicinal values** |
| --- | --- | --- | --- | --- | --- | --- |
| Cucurbitaceae | *Lagenaria siceraria* (Molina) Standl. | Qell (Am) | H | Fr | Homogenization [45,50] | Diarrhoea, vomiting, gonorrhea, wound, cancer, jaundice, intestinal parasites, dandruff , rabies, ear disease |
|  | *Mukia maderaspatana* (L.) M.J. Roem. | Gim-Hareg (Am) | H | R/S | Pounding and hanging [42] | Evil eye |
|  | *Peponium vogelii* (Hook.f.) Engl*.* | Surupa(Sd ) | Cl | L | Decoction [21] |  |
|  | *Zehneria scabra* (L. f.) Sond. | Areg-resa (Am) | Cl | R/L | Chewing [36, 69] | Anemia, febrile illness, headache, paralysis, external wound, mich, Eye infection, abdominal pain, ascariasis, unidentified swelling, dandruff, amenorrhoea, intelligence boost, wart, leprosy, wound dressing, measles, anthelmintic |
|  | *Cucumis ficifolius* A. Rich. | Yemdr Embuay (Am) | H | WP | Pounding and decoction[86] | Venereal diseases, rabies, wound, stomachache, amoebic dysentery, *“Mich”*, meningitis, evil eye, snake, bite, jaundice, tonsillitis, toothache, rheumatism, vomiting |
|  | *Cucumis dipsaceus* Ehrenb. ex Spach | Basu-bakule (Sd) | Cl | L | Infusion [21] | Snake bite, hepatitis |
|  | *Cucumis melo* L*.* | Hare-joge (Or) | H | R | Infusion [60] |  |
|  | *Momoridica foetida* Schumach | Achcha(Had) | H | L | [30] | Headache |
| Cyperaceae | *Cyperus distans* L.f. | Gebez-dhesha (Ham) | H | R/B | Pounding, concoction and infusion [22] |  |
| Ebenaceae | *Euclea divinorum* Hiern | Maqayta (Ko) | Sh | R | [23], concoction [54] |  |
|  | *Euclea racemosa* Murr. | Keleaw (Am) | Sh | R | [54, 59] | Snake bite, paralysis, abdominal pain, toothache, amoeba, evil eye, kidney problems |

| **Family** | **Scientific name** | **LN** | **Ha** | **PU** | **Methods of preparation(References)** | **Other medicinal values** |
| --- | --- | --- | --- | --- | --- | --- |
| Euphorbiaceae | *Jatropha curcas* L. | Habet-muluk (So) | Sh | S | Eating or concoction [86] | Headache, as purgative, Rabies |
|  | *Jatropha sp.* Friis | Atori(Af) | H | L | Pounding and homogenization [101] |  |
|  | *Albizia schimperiana* Oliv. | Maticho (Sd ) | Sh | L | Decoction [21] | Evil eye, swelling |
|  | *Andrachne aspera* Spreng. | Etse-tekeze (Am) | H | R | Concoction [54, 60] |  |
|  | *Acalypha indica* L. | Habrid(So) | H | AP | Pounding and decoction [86] | post-coital antifertility activity, snake bite |
|  | *Croton macrostachyus* Del. | Bissana (Am)  Bakanisa (Or) | T | L | Decoction[21], pounding and homogenization [28], decoction, concoction and roasting [31, 56], evaporating[44], squeezing[47], concoction and decoction [48, 66], smoking[49], concoction[69], decoction and concoction [88] | Rabies, intestinal parasites, Jaundice, skin diseases, gonorrhea, hemorrhage, gum ailments, febrile illness, headache, wound, dandruff, snake bite, urinary retention, blood clot, constipation , tetanus, venereal diseases |
|  |  |  |  | R | Decoction [55] |  |
|  |  |  |  | L/S | Decoction, squeezing, pounding and concoction [67] |  |
|  |  |  |  | SB | Pounding and concoction [68] |  |
|  |  |  |  | RB | Concoction [69], Pounding and concoction [87] |  |
|  |  |  |  | Sht | Concoction and cooking [77] |  |
|  | *Acalypha sp.* | Subaaci (Af) | H | WP | Pounding and infusion [100]. | Impotence, snakebite, head ache, arthritis, bleeding, wound |
|  | *Euphorbia abyssinica* Gmel*,* | Kulkual(Am)  Dharkena (Or) | H | Ne | Syrup [51] | Gastro- intestinal problems, rabies, hemorrhoids, skin diseases, jaundice |
|  |  |  |  | R | Pounding and concoction[67] |  |
|  |  |  |  | La | Homogenization and decoction [67], Concoction [75] |  |
| **Family** | **Scientific name** | **LN** | **Ha** | **PU** | **Methods of preparation(References)** | **Other medicinal values** |
| Euphorbiaceae | *Euphorbia schimperiana* Scheele | Bingele(Sd ) | Sh | L | Homogenization [21] |  |
|  | *Euporbia cactus* | Kalkalda(Tig) | Sh | La | Pounding and concoction[94] |  |
|  | *Phyllanthus maderaspatensis* L. | Harmel (Or) | H |  | [54] |  |
| Fabaceae | *Acacia etbaica* Schweinf. | Dere-kereta (Am) | T | L | [91] | Bronchitis, tonsillitis, gonorrhea, swelling, hemorrhoids, itching |
|  | *Indigofera articulata* Gouan | Gabalday (So) | Sh | R | Pounding, decoction and infusion [86] | Anaemia with jaundice, |
|  | *Indigofera coerulea* Roxb. | Habbi-hasey (So) | H | L | Pounding and decoction [86] | Wound dressing, constipation and wash against infected eyes |
|  | *Indigofera spicata* Forssk. | Shamtit(Am) | H | R | Chewing [38] | Diarrhea, cough, stomachache, toothache, retained placenta, evil eye, headache, goiter, wounds, intestinal parasites, meningitis |
|  | *Ajuga integrifolia* Buch.-Ham. ex D.Don | Anamuro (Sd), Tut-astil (Am) | H | AP | Decoction[21] | Intestinal parasites, epilepsy, wound, retained placenta, diarrhea, antifungal, hypertension, evil eye |
|  |  |  |  | WP | Squeezing [54, 98] |  |
|  | *Leucas sp.* | Blbalate (Sd) | H | AP | Homogenization [21] | Ascariasis, diarrhea, abdominal pain, urine retention, tonsillitis |
|  | *Albizia amara* (Roxb.) B.Boivin | Ondoddee (Kr) | T | B | Chewing [28] | *“Mich”*, cough |
|  | *Senna italica* Mill. | Sene-Meki(Am) | H | L | Pounding, decoction and concoction[86] Pounding and infusion [100] | Fever, jaundice, venereal diseases, allergy, stomach ache, snakebite worm expulsion |
|  | *Tamarindus indica* L. | Homar; Roqa (Am, So) | T | Fr | Concoction[51], infusion [53, 86, 104], | Stomachache/parasite, dysentery, wound, hemorrhoids fever |
|  | *Tephrosia gracilipes* Guill. & Perr. | Atotoka (Ku) | H | R | Pounding, homogenizing [92] |  |
|  | *Vigna unguiculata* (L.) Walp. | Epho-Gumuz (Or) | H | L, Se | Cooking [47] |  |
|  | *Zornia glochidiata* DC. | Halimi (Am) | H | R | Pounding , concoction and decoction [39] |  |

| **Family** | **Scientific name** | **LN** | **Ha** | **PU** | **Methods of preparation(References)** | **Other medicinal values** |
| --- | --- | --- | --- | --- | --- | --- |
| Fabaceae | *Acacia mellifera (Vahl.)* Benth*.* | Aygae (Kw) | T | B | Pounding and decoction [33] | Sexual incompetence of male, arthritis |
|  | *Acacia robusta Burch.* | Wangeyo (Or) | T | R | Concoction [51] |  |
|  | *Calpurnia aurea* (Ait.) Benth. | Chekata(Sd), Digita(Am) | Sh | L | Decoction[21], concoct ion, pounding, infusion [68], chewing [69], | Epidemics, internal diseases, swellings/ neck cancer, dysentery, hemorrhoids, rabies, diabetes, hypertension, excessive menstruation, fungal disease, external injury, mental disorder, urine retention, eye disease, snake bite, vomiting, impotency, jaundice |
|  |  |  |  | Se | Chewing [29] |  |
|  |  |  |  | Se &L | [66] |  |
|  | *Acacia seyal* Delile | Tundukiyac(Kr) | T | Gm | Chewing [28] | Epilepsy |
|  | *Cicer arietinum* L. | Shimbra (Am) | H | WP | Pounding and decoction [74] | Gastritis |
|  |  |  |  | Se  Fr/Se | Germinated and concoction [75]  Infusion and concoction [94] |  |
|  | *Entada abyssinica* (Steud. ex A. Rich.) Gilb. & Bout. | Ambalta (Or) | T | SB | Concoction and chewing. [57] |  |
| Flacourtiaceae | *Flacourtia indica* (Burm.f.) merr. | Akuku; Ququra (Am) | T | Fr | Eating [104] | Gastritis |
| Hydnoraceae | *Hydnora johannis* Becc. | Likke (So) | H | R | Pounding and decoction [86] | Diarrhea |
| Lamiaceae | *Leonotis ocymifolia* (Burm. f.) Iwarsson | Ferszeng, (Am) | Sh | Flr,  R, L | [42] | Diarrhea, Ascariasis, Febrile illness |
|  | *Leucas stachydiformis* (Hochst. ex Benth.) Briq. | Qumudu(Or) | H | WP | Vaporing [49] | Anaemia with jaundice |
|  | *Mentha spicata* L. | Nana (Am) | H | AP | Infusion or Bathing [86] | Common cold, headaches, digestive complaints |
| **Family** | **Scientific name** | **LN** | **Ha** | **PU** | **Methods of preparation(References)** | **Other medicinal values** |
| Lamiaceae | *Meriandra dianthera* (Roth, ex. Roem. & Schult.) Briq. | Mesaguh(Tig ) | T | L | Pounding and squeezing [87] | Hypertension, diarrhea |
|  | *Ocimum spicatum* Deflers | Shero(So) | H | L | Decoction[86] | Tenia versicolor |
|  | *Ocimum basilicum* L. | Ajuban(Am) | H | L | Chewing [42,99], Pounding [45] | Headache, abdominal pain |
|  | *Ocimum canum* Sims | Biyangua(Am) | Sh | Flr | Cooking [103] |  |
|  | *Ocimum gratissimum* L. var. gratissimum | Damakase(Am) | H | L | Pounding [69] | Alergic |
|  | *Ocimum lamiifolium* Hochst.ex Benth | Damakessie (Am) | T | AP, B | Concoction [21] | *“Mich”*, antibacterial, antifungal, insecticidal, antipyretic, headache, evil eyes, cutaneous leishmaniasis , febrile , tonsillitis, cough |
|  | *Ocimum urticifolium* Roth. | Angabisha(Sd) | Sh | L | Concoction [21] | Toothache, oral thresh, ear infection, mouth infection, tongue infection, michi |
|  | *Otostegia integrifolia* Benth. | Tinjut(Am) | Sh | L | Pounding [61, 69] | “mich”, *“megagna*”, evil eye, ascariasis, abdominal pain, lice or fleas infestation, common cold |
|  |  |  |  | L& Sht | Pounding, concoction [68] |  |
|  | *Premna oligotricha* Baker |  | Sh | L | Pounding and homogenization [39] |  |
|  | *Premna schimperi* Engl. | Udo(Sd), Checho(Am) | Sh | L | Homogenization[21] | Hemorrhoids, wound, hypertension, inflammation of skin, Eye Sickness, toothache |
|  | *Satureja punctata* (Benth.) Briq. | Amessa(Sd ) | H | AP | Homogenization[21] |  |
|  | *Clerodendrum myricoides* (Hochst.) Vatke | Misrich(Am), Madesisa(Sd) | Sh | L | Infusion [21] | Urine retention, snake and spider poison, headache, evil eye, diarrhea, stomachache, viral infection |
|  |  |  |  | R | Pouning/ homogenizing [52, 59, 64, 61, 68] |  |
|  | *Plectranthus punctatus* subsp. edulis (Vatke) Morton | Sheshe dinikata(Ke) | H | L | [24] | Epilepsy, rheumatism, evil eye, pertussis |
|  | *Pycnostachys abyssinica* Fresen | Tontona(Ke) | Sh | R | [24] | Eye ache, diarrhea, cholera, ascraris, abdominal complaints, antiemetic |
| **Family** | **Scientific name** | **LN** | **Ha** | **PU** | **Methods of preparation(References)** | **Other medicinal values** |
| Loganiaceae | *Nuxia congesta* R.Br. ex Fresen. | Nole(Sd ) | Sh | L | Infusion [21] |  |
|  | *Buddleja polystachya* Fresen. | Askuar(Am) | Sh | R/L/B | Pounding [32] | Epilepsiy; stomachache |
| Lythraceae | *Lawsonia inermis* L. | Hinna (Am) | T | R / B | Pounding, squeezing and decoction [86] | Fungal infections |
| Malvaceae | *Gossypium barbadense* L. | Tit (Am) | Sh | Se | Infusion[66] |  |
|  | *Gossypium hirsutum* L*.* | Titt (Am) | H | Se | Pounding and infusion [83] |  |
|  | *Sida schimperi*Hochst. ex A. Rich. | Chifrig(Am) | Sh | Se | Pounding and homogenization [65] |  |
| Melanthiaceae | *Bersama abyssinica* Fresen. | Oloncho (Sd) | Sh | L  WP | Homogenization[21]  Decoction[62] | Syphilis, ascariasis, dysentery, wound, tonsillitis, retained placenta, rabies, bronchitis, febrile illness |
| Meliaceae | *Melia azedarach* L. | Neem-zaf(Am) | T | Tw/ Sht | Homogenization[21], squeezing [29] | Diarrhea, headache, toothache, intestinal parasites, wound, acute bleeding, tetanus, dandruff, tonsillitis |
|  |  |  |  | L | Pounding and infusion[27], squeezing[49,87], decoction [104] |  |
|  |  |  |  | Se | Squeezing [47] |  |
|  | *Turraea mombassana* Hiern ex C. DC. | Piterkama (Am) | Sh | R | [23] |  |
|  | *Azadirachta indica* A. JUSS. | Kinin (Am) | T | L | [51, 102], squeezing and infusion [53], concoction and chewing [69], pounding and infusion [86] | Dysentery, intestinal parasite, fungal infection, wound |
|  |  |  |  | Se &L | Concoction [102] |  |

| **Family** | **Scientific name** | **LN** | **Ha** | **PU** | **Methods of preparation(References)** | **Other medicinal values** |
| --- | --- | --- | --- | --- | --- | --- |
| Menispermaceae | *Stephania abyssinica* (Dillon & A. Rich) | Kalala(Sd), Yeait-hareg (Am) | Cl | R | Infusion [21] | Wound, impotence, rabies, syphilis, diarrhea, jaundice, ascariasis, stomachache, retained placenta |
|  | *Cissampelos pareira* L*.* | Mile (Kw) | Cl | R | Pounding/infusion[33] | Chest pain/ TB/ coughing with blood, swelling, stomachache, diarrhea, febrile disease, intestinal parasites |
|  | *Cissampelos mucronata* A.Rich. in Guill Perrot. & A.Rich | Migaar (Or) | Cl | L&R | [59] | Dysmenorrhagia, menorrhagia, infertility |
| Moraceae | *Dorstenia barnimiana* Schweinf. | Werk-bemieda (Am) | H | R | Infusion [84] | Hemorroid, cancer, *“Ejeseb”*, evil sprit |
|  | *Ficus platyphylla* Del. | Hadawa (Ko) | T | SB | Pounding and concoction [23] |  |
|  | *Ficus sur* Forssk. | Shola(Am) | T | Fr, | Pounding and concoction[27] | Wound, acute bleeding, vomiting |
|  |  |  |  | La | [67] |  |
|  | *Ficus vasta* Forssk | Warka(Am) | T | B | [91] | Skin itching, injury, diarrhea, rabies, tonsillitis |
| Moringaceae | *Moriga oleifera* | Shiferaw (Am) | T | L | Pounding, decoction and infusion [100]. | Amoebiasis |
|  | *Moringa stenopetala*(Bak. f.) Cuf. | Shiferaw (Am) | T | L/R | Homogenization [21], decoction, infusion and , concoction [22], decoction[23, 32] | Hypertension, stomach pain, retained placenta, flu, jaundice ,vomiting |
| Myrsinaceae | *Maesa lanceolata* Forssk. Suwaria | Abayi(Or); Kalawa (Am) | T | Fr | Eating [54] | Leprosy |
| Myrtaceae | *Syzygium guineense* (Willd.) DC. | Duwancho (Kr) | T | L | Concoction [28] | Snake bite, gonorrhea, hemorrhoid, internal worms |
|  | *Eucalyptus globulus* Labill. | Nech-bahirzaf (Am) | T | L | Evaporating[21, 99], Smoked[49] decoction [29, 85] | Bronchitis, sore throat, viral infections, skin diseases, *“Mich”*, typhoid, acute sickness, cough, ascariasis |
| Olacaceae | *Ximenia americana* L*.* | Hudha(Or) | Sh | B | Decoction [63] | Snake bite, hepatitis, swelling of the pancreas, |

| **Family** | **Scientific name** | **LN** | **Ha** | **PU** | **Methods of preparation(References)** | **Other medicinal values** |
| --- | --- | --- | --- | --- | --- | --- |
| Oleaceae | *Jasminum abyssinicum .*ex Delile | Tembelel (Am) | Cl | S | Pounding and decoction [66] | Wound, snake bite, tape worm, vomiting, tonsilities, toothache, rabies |
|  | *Olea europaea L.* | Girari(Su)  Woyra(Am) | T | B | Pounding and homogenization [40]  Infusion[88] | Abdominal pain, dysentery, anti-helminthes, irritation of eye, brain tumor |
| Papaveraceae | *Argemone mexicana* L. | Kolcolich (Sd ) | Sh | R | Decoction [21] | Wound, leshimaniasis, bloody urine, diarrhea, intestinal parasites |
| Pedaliaceae | *Sesamum indicum* L. | Eshkulubia (Ku) | H | R | Pounding and decoction [92] | Snake bite |
| Phytolaceae | *Phytolacca dodecandra* L'Herit | Mekan-endod (Am) | Sh | L | Pounding and homogenization [31, 32, 56, 71], squeezing[75] | Abdominal pain, toothache , gonorrhea, rabies, abortion, itching, wound, hepatitis, jaundice, elephantiasis |
|  |  |  |  | L&R | [66] |  |
|  |  |  |  | B | Pounding and infusion [67] |  |
|  |  |  |  | WP | Infusion[88] |  |
| Plumbaginaceae | *Plumbago zeylanica* L. | Warwaro  (Ka, Kw),  Telba(Am) | H | R | Pounding/decoction [33, 59] | Chest pain and coughing with blood/TB/, tiredness, fever, swelling, febrile disease, rheumatism, diarrhea, abscess, abortion, retained placenta, anti-bacterial ,cancer, hypertension, TB, impotence, heart disease, hemorrhoids |
|  |  |  |  | L | Chewing [69] |  |
| Poaceae | *Elucine coracana* (L.) Gaertn. | Dagusa (Am) | H | Se | Infusion & fermented [71] |  |
| Polygalaceae | *Securidaca longipedunculata* Fresen. | Etsamanaay (Am) | T | R | Pounding, concoction and smoking [73] | Gonorrhea, syphilis, evil spirits, *“Mich”*, abdominal problems |
| Polygonaceae | *Rumex abysinicus* | Mekimeko (Am) | H | R | Decoction [72] | Stomachache, wound, hypertension, common cold and ring worms |
|  |  |  |  | L | Pounding and decoction [65] |  |
|  | *Rumex nervosus*Vahl | Embacho (Am) | Sh | L | Concoction [42] | Scabies, Abdominal pain, rabies |

| **Family** | **Scientific name** | **LN** | **Ha** | **PU** | **Methods of preparation(References)** | **Other medicinal values** |
| --- | --- | --- | --- | --- | --- | --- |
| Ranunculaceae | *Clematis hirsuta* Perr. & Guill. | Fede(Sd), Azo-hareg(Am)  Jibija (Gu) | H | L | Homogenization[21] | Wound, trachoma, elephantiasis, hemorrhoids, gastro-intestinal complaints, respiratory tract problem, cataract |
|  | *Clematis simensis* Fresen | Sido(Sd ) Azo-hareg (Am) | Cl | L | Decoction[21], squeezing[103] | Wound, lung TB, leprosy, syphilis, eye infection, swelling, headache |
|  |  |  |  | R | Pounding [92] |  |
|  | *Nigella sativa*L*.* | Tiqur-Azmud(Am) | H | Fr , R | Pounding and concoction [25, 44] | Abdominal colic, pneumonia, headache |
| Rhamnaceae | *Ziziphusmauritiana* Lam*.* | Kurkura (Am) | T | Se | Pounding and homogenization [104] | Diarrhea, wound |
| Rosaceae | *Prunus persica* (L.) Batsch | Kok(Am) | T | Se | Pounding [69] | Appetite, Swelling |
|  | *Rosa abyssincia* Lindley. | Qega(Am) | Sh | Fr | [91] | Tapeworm, stomachache |
|  | *Hagenia abyssinica* (Bruce) J. F. Gmelin | Koso (Am) | T | R | Decoction [55], concoction [54] | Stomachache, diarrhea, tape worm, general illness |
|  |  |  |  | SB | Pounding and concoction [83] |  |
| Rubiaceae | *Rubia cordifolia* L. | Hare(Sd) | Cl | R | Chewing [21] | Coughing |
|  | *Canthium pseudosetiflorum* Bridson | Medhel(Ham) | Sh | L | Pounding and infusion [22] | Vomiting |
|  | *Coffea arabica* L. | Buna(Am) | Sh | WP | Smoking[27] | Gastritis, headache, sudden sickness |
|  | *Gardenia lutea* Fresen | Gambelo (Shn) | T | R | Homogenization [69] |  |
|  | *Gardenia ternifolia* Schumach. & Thonn | Bodut(Mnt) | T | SB | Chewing[38] | Hepatitis, evil eye, allergy |
|  |  |  |  | R | Pounding and infusion [55] |  |
|  | *Pavetta abyssinica* Fresen | Fugi-Miqichut (Ke) | Sh | Se | [24] | Rheumatism, evil eye, Epistaxis, epilepsy |

| **Family** | **Scientific name** | **LN** | **Ha** | **PU** | **Methods of preparation(References)** | **Other medicinal values** |
| --- | --- | --- | --- | --- | --- | --- |
| Rutaceae | *Ruta chalepensis* L. | Sunkuruta(Sd )  Tena-Adam (Am) | Sh | Tw  L | Decoction[21, 68]  Homogenization [28], Pounding [87] | Stomachache, ‘*Megagn*a’, headache, cold, evil eye, cough, hemorrhoids, fever, *'Dingetega'* , toothache , *“Mich”* |
|  | *Vepris glomerata* (F. Hoffm.)Engl. | Kena(Ham) | Sh | B/L | Pounding and infusion [22] | Abdominal colic |
|  | *Fagaropsis anolensis* (Engl.) Dale | Godecho (Sd ) | T | Se | Chewing [21] | Stomach-ache, gonorrhea |
| Salvadoraceae | *Salvadora persica* L. | Yeharer Mefaqya (Am) | H | R | Chewing[22], pounding and decoction [33], Pounding and infusion [100], | Oral hygiene (antibacterial), abscess, TB /coughing with blood, flu, febrile disease, cancerous swelling, diarrhea/stomach-ache, hypertension, tonsillitis |
|  |  |  |  | L | Pounding and decoction[86] |  |
| Santalaceae | *Osyris quadripartita* Decn. | Karicho(Sd ) | Sh | L&R | [10] | Leprosy, leishmaniasis, cough, rabies, schistosomiasis, abdominal pain, urine problem |
|  |  |  |  | L | Infusion [21] |  |
| Sapindaceae | *Dodonaea angustifolia* L*.*f | Kitkita (Am) | T | Fr | Eating [66, 95, 102] | Eye infection, burn, intestinal parasite, dandruff, rabies, wound, bone fracture |
|  |  |  |  | L&Fr | Pounding, concoction[68, 102] |  |
|  |  |  |  | Se | Pounding and concoction [88], [69] |  |
| Simaroubaceae | *Brucea antidysenterica* J.F.Mill. | Hatawicho(Sd) Aballo(Am) | T | L,S&B | Infusion[21], decoction [62], concoction [72] | Stomachache, evil eye, hepatitis, wound, dermatophytes, cancer, venereal diseases, helimethiasis, snake bite, tooth ache, jaundice, eczema, rheumatism, rabies |
|  |  |  |  | Se | Eating [45] |  |
|  |  |  |  | R /L | Chewing [57] |  |
|  | *Harrisonia abyssinica* Oliv. | Moy Moy (Kw) | T | B& R | Pounding /infusion/decoction[33] | Stomachache, loss of appetite, vomiting, coughing with blood |

| **Family** | **Scientific name** | **LN** | **Ha** | **PU** | **Methods of preparation(References)** | **Other medicinal values** |
| --- | --- | --- | --- | --- | --- | --- |
| Solanaceae | *Lycium shawii* Roem and Schult | Hedalusayto (Af) | Sh | R | Pounding and infusion [100] |  |
|  | *Nicotianatobaccum* L. | Timbaho (Am) | H | R | Concoction [29] | Swelling |
|  | *Solanum incanum* L. | Chucho(Sd), Embuay (Am) | Sh | L | Infusion[21] | Chest pain, dysentery, stomachache, irritation, toothache, bleeding, wound, tonsillitis, *“mich*”, lymphangitis, snake bite, gonorrhea, ear pain |
|  |  |  |  | R | [59] |  |
|  | *Withania Somnifera* L. Dunal | Gizawa(Am) | Sh | L | Homogenization [21], squeezing [48] | Arthritis, chest pain, evil eyes, headache, common cold, tonsillitis, vomiting, dislocated bone, eye infection, *“mich”*, itching, paralysis, diarrhea, , swelling, hepatitis, stomachache |
|  |  |  |  | L&R | [23] |  |
|  |  |  |  | B&L | [59] |  |
|  |  |  |  | Se | Pounding and homogenization[65] |  |
|  |  |  |  | R | Smoking[73], decoction[86], pounding, concoction, |  |
|  | *Capsicum annuum* L. | Mitmita(Am) | H | Fr | Concoction [69] , pounding and concoction or infusion [75, 83] | Appetite loss, stomach-ache, headache |
|  | *Datura stramonium* L*.* | Manjii(Or) | H | Fr | Pounding and concoction [50] | Dandruff, headache, coughing |
|  | *Solanum dasyphyllum* Schumach. | Geber-Emboy (Am) | H | R | Pounding and homogenization [65] |  |
|  | *Solanum hastifolium*  Hochst. ex Dunal | Lodocamurae (KA), Dinkishea(KW) | Sh | R | Chewing/crushing/infusion[33] | Diarrhea, amoebic dysentery with blood,  stomach-ace |
| Thymelaeacea | *Gnidia stenophylla* Gilg. | Demerarit (Am) | H | R | [59] | Ascaris, rabies |
|  | *Gnidia involucrate* Steud ex.A.Rich*.* | Demerarit (Am) | H | R/L | Pounding, homogenization [66, 68] | Abdominal pain, syphilis, wound, cancer, STDS, TB, mental problems |
| Tiliaceae | *Grewia ferruginea* Hochst. ex A. Rich | Lenquata(Or) | Sh | R | Concoction[54] | Retained placenta, eye disease |

| **Family** | **Scientific name** | **LN** | **Ha** | **PU** | **Methods of preparation(References)** | **Other medicinal values** |
| --- | --- | --- | --- | --- | --- | --- |
| Urticaceae | *Droguetia iners* (Forssk.) Schweinf. | Yewoba-medihanit (Am) | H | L | Pounding and decoction [39] |  |
|  | *Girardinia diversifolia*(Link) Friis | Hiddi (Or) | H | R | Concoction[41] | Skin disease |
| Verbenaceae | *Lantana camara* L*.* | Yewof-kolo (Am) | Sh | L | Decoction [29] | Fibril illness |
|  | *Lantana trifolia* L. | Yewof-kolo (Am) | Sh | L  R | Pounding and decoction [32]  Pounding, infusion and homogenization [39] | Sight problem of eye, heart tired, |
|  | *Lippia adoensis* Hochst. ex Walp var adoensis | Koseret (Am) | Sh | WP | [54] |  |
| Vitaceae | *Cissus rotundifolia* (Forssk).  Vahl | Chambie (Am) | Cl | R, B | Pounding and decoction [86] | Gonorrhea , dysentery, stomachache, coughing with blood/TB/, rheumatism |
|  | *Cyphostemma niveum* (Hochst. ex Schweinf.) | Dashe(Sd ) | Cl | L | Infusion[21] |  |
| Zingiberaceae | *Ginger officinale*Roscoe | Ginger(Am) | H | Rh | Pounding and concoction [48] | abdominal cramps, common  cold, tonsillitis |

_LN= Local Name (Am = Amaharic, Or = Afaan Oromo, Sd = Sidama , Tig=Tigrrigna, So= Somali, Kr=Koorete, Af= Afar, Ham= Hamer, Gu= Gumuz, Ko=Konso, Shk=Sheko, Shn=Shinasha, Mnt=Meinit, Su= Suri, Ku= Kunama, Kw= Kwego, Ka=Kara, Ke=Kembatissa); Ha= Habit( T=Tree, H=Herb, Sh=Shrub, Cl= Climber); PU= Part Used(L=Leaf, S=Stem, R=Root, B=Bark, RB=Root Bark, SB=Stem Bark, Gm=Gum, AP= Arial Part, WP=Whole Part, Bu= Bulb, Fr=Fruit, La=Latex, Exd=Exudate, Se=Seed, Sht= Shoot, Tw= Twig, Ne=Nectar, RE= Root Exudate, Fl=Fluid, Flr= Flower, Rh=Rhizome)_
